# Rare symptom of left inguinal abscess secondary to a retroperitoneal perforation of diverticulitis of the sigmoid colon: A case report

**DOI:** 10.1097/MD.0000000000039770

**Published:** 2024-09-27

**Authors:** Mio Nihei, Teppei Kamada, Takashi Aida, Daisuke Yamagishi, Junji Takahashi, Keigo Nakashima, Eisaku Ito, Norihiko Suzuki, Taigo Hata, Masashi Yoshida, Hironori Ohdaira, Yutaka Suzuki

**Affiliations:** a Department of Surgery, International University of Health and Welfare Hospital, Iguchi, Nasushiobara, Tochigi, Japan.

**Keywords:** case report, diverticulitis, inguinal abscess, retroperitoneal perforation

## Abstract

**Rationale:**

Complicated colorectal diverticulitis could be fatal, and an abscess caused by this complication is usually formed at the pericolic, mesenteric, or pelvic abscess. Therefore, we report a rare case of sigmoid colon diverticulitis that developed a large inguinal abscess.

**Patient concerns:**

A woman in her 70s was admitted to our hospital with a chief complaint of left inguinal swelling and tenderness 1 week before admission. Physical examination showed swelling, induration, and tenderness in the left inguinal region. Blood tests revealed elevated inflammatory reaction with C-reactive protein of 11.85 mg/dL and white blood cells of 10,300/μL. Contrast-enhanced computed tomography showed multiple colorectal diverticula in the sigmoid colon, edematous wall thickening with surrounding fatty tissue opacity, and abscess formation with gas in the left inguinal region extending from the left retroperitoneum.

**Diagnoses:**

The diagnosis was sigmoid colon diverticulitis with large abscess formation in the left inguinal region.

**Interventions:**

Immediate percutaneous drainage of the left inguinal region was performed, as no sign of panperitonitis was observed. Intravenous piperacillin-tazobactam of 4.5 g was administered every 6 hours for 14 days.

**Outcomes:**

The inflammatory response improved, with C-reactive protein of 1.11 mg/dL and white blood cell of 5600/μL. Computed tomography of the abdomen confirmed the disappearance of the abscess in the left inguinal region, and complete epithelialization of the wound was achieved 60 days after the drainage. The patient is under observation without recurrence of diverticulitis.

**Lessons:**

We report a rare case of sigmoid colon diverticulitis that developed a large inguinal abscess, which was immediately improved by percutaneous drainage and appropriate antibiotics administration.

## 1. Introduction

Colorectal diverticulitis is a common disease, and its recent prevalence has increased treatment opportunities.^[1,2]^ Diverticular hemorrhage and diverticulitis are common complications of colorectal diverticulitis, and the incidence of diverticulitis is 3 times higher than that of diverticular hemorrhage.^[3]^

Colorectal diverticulitis is divided into 2: simple and complicated. Complicated diverticulitis is defined as diverticulitis with perforation, abscess, obstruction, and fistula, leading to peritonitis or septic shock, and could be fatal.^[4]^

Based on Hinchey’s classification, complicated diverticulitis indicates that an abscess is usually formed at the pericolic, mesenteric, or pelvic abscess. The size and location of the abscess determine the treatment. Diverticulitis is treated conservatively when the diameter of the abscess is 3 cm or less. Surgical drainage is considered when the diameter of the abscess exceeds 5 cm.^[5]^ Patients with diverticulitis with perforation or abscess often present with fever, abdominal pain, and peritonitis. No colorectal diverticulitis with symptoms of inguinal swelling owing to abscess formation at the inguinal region has been reported.

We report a rare case of sigmoid colon diverticulitis that developed a large inguinal abscess, which was immediately improved by percutaneous drainage and antibiotic administration.

## 2. Patient information

A woman in her 70s was admitted to our hospital with a chief complaint of left inguinal swelling and tenderness 1 week before admission. Her medical history was ischemic colitis and acute appendicitis. She smokes 10 cigarettes per day. Her vital signs were stable, and she was afebrile.

## 3. Clinical Findings

**Figure 1.**
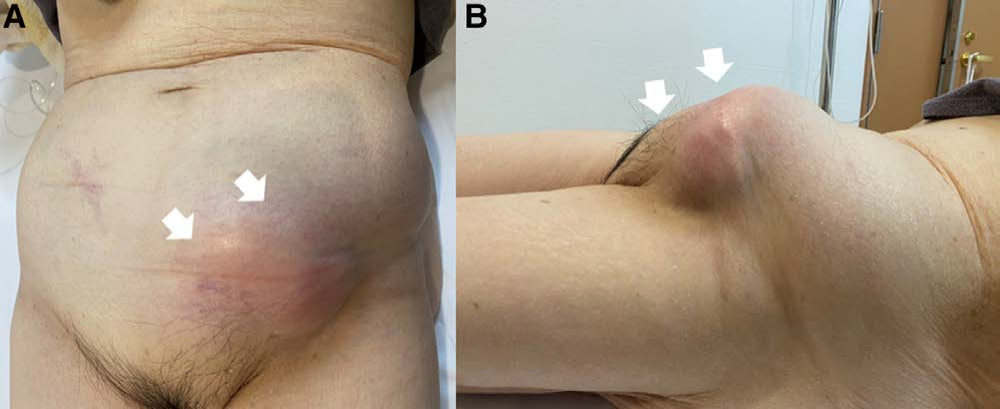
Left inguinal swelling and tenderness on the day of admission. (A) Frontal view (arrows). (B) Lateral view (arrows).

On physical examination, the abdomen was soft and flat, with no signs of peritonitis. Swelling, induration, and tenderness were observed in the left inguinal region (Fig. 1A and 1B).

## 4. Diagnostic Assessment

**Figure 2.**
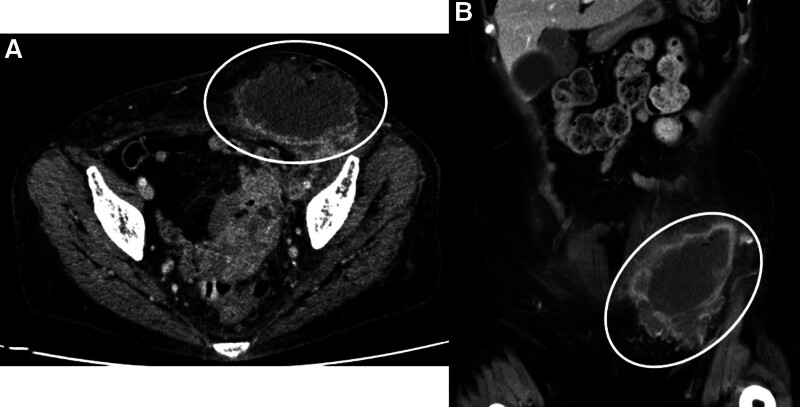
Contrast-enhanced computed tomography showed large abscess formation with gas in the left inguinal region extending from the left retroperitoneum. (A) Coronal image (circle). (B) Sagittal image (circle).

Blood tests revealed elevated inflammatory reaction with C-reactive protein of 11.85 mg/dL and white blood cell of 10,300/μL (neutrophil, 81.4%). Contrast-enhanced computed tomography (CT) showed multiple colorectal diverticula in the sigmoid colon, edematous wall thickening with surrounding fatty tissue opacity, and abscess formation (the maximum diameter of 85 mm) with gas in the left inguinal region extending from the left retroperitoneum (Fig. 2A and 2B). Colorectal malignancy was denied following the results of a lower colonoscopy performed within a year. The patient was hospitalized on the same day with a diagnosis of sigmoid colon diverticulitis with a large abscess formation in the left inguinal region.

## 5. Therapeutic intervention

Percutaneous drainage of the left inguinal region was performed, as no sign of panperitonitis was observed. An approximately 5-cm incision along the skin break line was made under local anesthesia, and a large amount of white, malodorous, purulent fluid was drained. The wound was washed with 1 L of isotonic sodium chloride solution, and hemostasis was achieved using gauze packing and drainage. β-streptococcus group F and *Bacteroides fragilis* were detected in the drainage fluid culture.

The patient was hospitalized and managed with fasting and intestinal rest. Intravenous piperacillin-tazobactam of 4.5 g was administered every 6 hours for 14 days. The patient was given food on the seventh day after admission.

## 6. Follow-up and outcomes

The inflammatory response improved, with C-reactive protein of 1.11 mg/dL and white blood cell of 5600/μL (neutrophil 67.1%). The amount of discharge from the wound decreased; hence, the patient was managed as an outpatient and instructed to continue self-wound washing at home.

**Figure 3.**
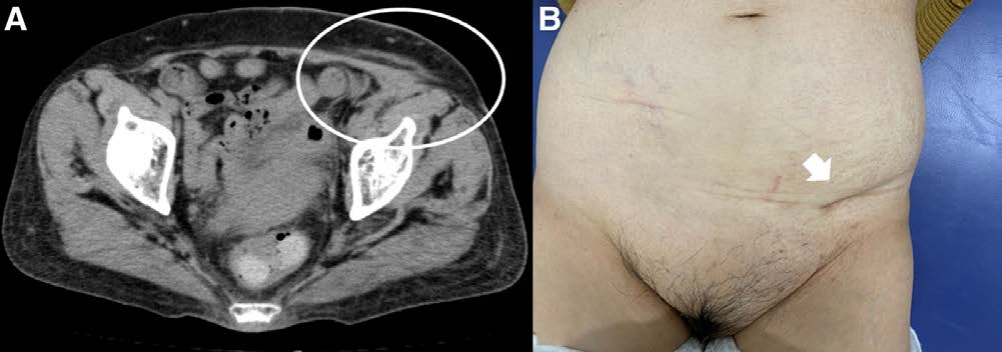
(A) Computed tomography confirmed the disappearance of the abscess in the left inguinal region (circle). (B) Complete epithelialization of the wound on the 60th day after the drainage (arrow).

On the 60th day after the drainage, CT of the abdomen confirmed the disappearance of the abscess in the left inguinal region, and complete epithelialization of the wound was achieved (Fig. 3A and 3B). The patient is under observation without recurrence of diverticulitis.

## 7. Discussion

We report an unusual symptom of large left inguinal abscess formation owing to retroperitoneal perforation in a patient with sigmoid diverticulitis.

Right-sided colonic diverticulitis is more common in patients younger than 60 years, whereas left-sided colonic diverticulitis is more common in older patients.^[6,7]^ Smoking and obesity are risk factors for exacerbation of diverticulitis complications.^[8,9]^ The mortality rate of complicated diverticulitis with complications such as retroperitoneal abscess, fistula formation, and perforation is 2.8%; hence, complicated diverticulitis could be fatal.^[6]^ Complicated diverticulitis usually presents with high fever, abdominal pain, and pneumaturia.

Abscesses commonly form in the retroperitoneal or pelvic region.^[5]^ Some case reports have demonstrated that inflammation from a retroperitoneal abscess, developed via perforation of colorectal diverticulitis, can spread to the brain or cause mediastinum emphysema.^[10,11]^ Additionally, complicated diverticulitis presenting with a chief complaint of inguinal distension can cause abscesses localized in the inguinal area.

This case showed that a large abscess extending from retroperitoneal perforation to the inguinal region could be managed using percutaneous drainage and antimicrobial therapy without surgery. The mechanism of the spread of the retroperitoneal abscess to the inguinal region may be attributed to anatomical features. The retroperitoneal space, a part of extraperitoneal space, extends from the subdiaphragm to the posterior part of the trunk in the pelvic region, situated between the transversalis fascia and parietal peritoneum. This space is divided into 3 compartments (anterior pararenal, perirenal, and posterior pararenal space, respectively) via the anterior renal fascia, posterior renal fascia, and lateroconal fascia. The posterior pararenal space is in contact with the psoas major muscle on the inside and is continuous with the preperitoneal fat layer on the outside, sandwiched between the transversalis fascia and the parietal peritoneum in the fat layer outside the lateral conical fascia.^[12]^ As the pelvic muscles, such as psoas major and iliac muscles, arise from the lumbar spine and attach to the femur, we considered that retroperitoneal inflammation developed along these fascia via the dorsal side of the inguinal ligament. The inflammation spreads from the preperitoneal fat layer via the inguinal canal to the left anterior inguinal region.

In this case, we considered 2 reasons for the immediate improvement achieved. First, the inguinal abscess was in a position where percutaneous drainage was possible. Second, the causative bacterium, β-streptococcus and *Bacteroides fragilis*, including gram-positive cocci, gram-negative bacilli, and anaerobic bacteria, were effectively covered by the spectrum of piperacillin-tazobactam.

Furthermore, checking for inguinal hernia complications using CT before percutaneous drainage is important to avoid damage to other organs.

## 8. Conclusion

We experienced a rare case of sigmoid colon diverticulitis that developed a large inguinal abscess, which was immediately improved using percutaneous drainage and appropriate antibiotics administration. Sigmoid colon diverticulitis with retroperitoneal abscess confined to rare locations should be managed using appropriate surgical drainage and antibiotic administration.

## Author contributions

**Conceptualization:** Mio Nihei, Teppei Kamada.

**Data curation:** Mio Nihei, Takashi Aida, Daisuke Yamagishi, Junji Takahashi, Keigo Nakashima, Eisaku Ito, Norihiko Suzuki, Taigo Hata, Masashi Yoshida, Hironori Ohdaira.

**Writing – original draft:** Mio Nihei.

**Writing – review and editing:** Teppei Kamada, Yutaka Suzuki.
